# Induction of antigen-specific immune responses in mice by recombinant baculovirus expressing premembrane and envelope proteins of West Nile virus

**DOI:** 10.1186/1743-422X-9-132

**Published:** 2012-07-16

**Authors:** Bibo Zhu, Jing Ye, Ping Lu, Rong Jiang, Xiaohong Yang, Zhen F Fu, Huanchun Chen, Shengbo Cao

**Affiliations:** 1 State Key Laboratory of Agricultural Microbiology, Huazhong Agricultural University, Wuhan, Hubei, 430070, People's Republic of China; 2 Laboratory of Animal Virology, College of Veterinary Medicine, Huazhong Agricultural University, Wuhan, Hubei, 430070, People's Republic of China; 3 Department of Pathology, University of Georgia, Athens, GA, 30602, USA; 4 China Animal Health and Epidemiology Center, Qingdao City, Shandong Province, 266032, People's Republic of China

**Keywords:** West Nile virus, Rcombinant baculovirus, Premembrane/Envelope protein, Immune responses

## Abstract

**Background:**

West Nile Virus (WNV) is an emerging arthropod-born flavivirus with increasing distribution worldwide that is responsible for a large proportion of viral encephalitis in humans and horses. Given that there are no effective antiviral drugs available for treatment of the disease, efforts have been directed to develop vaccines to prevent WNV infection. Recently baculovirus has emerged as a novel and attractive gene delivery vehicle for mammalian cells.

**Results:**

In the present study, recombinant baculoviruses expressing WNV premembrane (prM) and envelope (E) proteins under the cytomegalovirus (CMV) promoter with or without vesicular stomatitis virus glycoprotein (VSV/G) were constructed. The recombinant baculoviruses designated Bac-G-prM/E and Bac-prM/E, efficiently express E protein in mammalian cells. Intramuscular injection of the two recombinant baculoviruses (at doses of 10^8^ or 10^9^ PFU/mouse) induced the production of WNV-specific antibodies, neutralizing antibodies as well as gamma interferon (IFN-γ) in a dose-dependent pattern. Interestingly, the recombinant baculovirus Bac-G-prM/E was found to be a more efficient immunogen than Bac-prM/E to elicit a robust immune response upon intramuscular injection. In addition, inoculation of baculovirus resulted in the secretion of inflammatory cytokines, such as TNF-α, IL-2 and IL-6.

**Conclusions:**

These recombinant baculoviruses are capable of eliciting robust humoral and cellular immune responses in mice, and may be considered as novel vaccine candidates for West Nile Virus.

## Background

West Nile Virus (WNV), a mosquito-born virus, belongs to the Japanese encephalitis serogroup of flaviviruses [1] and is an outstanding example of a zoonotic pathogen as its geographic range spans North and South America, Europe, the Middle East, Africa, Western Asia and Australia [2,3]. Zoonotic transmission of WNV involves Culex mosquitos and birds as the natural reservoir hosts, with humans and horses as dead-end hosts [4]. WNV infections are ranging from a sub-clinical illness, to a self-limiting febrile syndrome or a lethal neuroinvasive disease, and the incidence of severe disease and death increases with age [5,6]. Since the emergence of WNV in north American in 1999, WNV has been responsible for over 12,000 cases of meningitis/encephalitis, and over 1,100 fatalities in humans and mass mortality of resident birds [7]. The arbovirus infection of humans and animals is becoming a major public health and veterinary concern [4]. The WNV outbreak in North America coupled with the absence of specific therapy against WNV infection has triggered vaccine development to prevent the infection of humans and animals. It has been shown that virion envelope is embedded with viral envelope (E) and premembrane/membrane (prM/M) proteins. The prM protein helps proper folding of the E protein by forming heterodimers [8] and is cleaved into M by furin [9,10]. The glycoprotein E is responsible for many properties of the virus including host range, replication, assembly, and stimulation of humoral and cellular immune responses [11]. A variety of WNV vaccine candidates based on the glycoprotein E, which evokes the majority of the neutralizing antibodies, have been developed. Three equine vaccines have been successfully commercialized in the USA, including Innovator^TM^, formulated based on formalin-inactivated virus, Recombitek^TM^, a recombinant replicative canarypoxvirus vaccine, and pCBWN, a recombinant plasmid DNA vaccine [12]. Novel recombinant subunit vaccine candidates such as virus-like particles (VLPs), vectoring vaccine candidates, DNA vaccine candidates [12], and TripliVAX JE, a chimeric vaccine expressing prM/E and NS1 gene of Japanese encephalitis virus [13], have shown protective immunity in animal model. However, there are no vaccines currently available for humans. With the growing volume of international travel and commerce, exotic pathogens can spread quickly between continents. Therefore, safe and effective vaccines of WNV are needed for humans.

In recent years, baculovirus has emerged as a vector for gene delivery and vaccine development, with promising newcomer being Autographa californica multiple nucleopolyhedrovirus (AcMNPV), a member of baculoviridae family. The host specificity of baculovirus was originally thought to be restricted to cells derived from a few taxonomically related insect species [14]. However, it has been shown to infect a number of mammalian cells without replication [15-18]. Additionally, accumulated evidence has revealed that baculovirus express foreign proteins not only in insect cells, but also in mammalian cells under the transcriptional control of mammalian promoters [14,16,19]. Subsequently, it has been reported that a baculovirus pseudotyped with the glycoprotein (G) of the vesicular stomatitis virus (VSV/G) appeared to help the virus escape from the endosomes, which increases transduction efficiency in mammalian cells [20,21]. Furthermore, the baculovirus has advantages of being a strong adjuvant and thus can induce inflammatory cytokines and interferons [22-24]. To this end, several studies have demonstrated that direct vaccination with recombinant baculovirus can induce high-level humoral and cell-mediated immunity against various antigens, including pseudorabies virus glycoprotein B (gB) [25], GP5 and M protein of porcine reproductive and respiratory syndrome virus (PRRSV) [26], capsid protein of procine circovirus type2 [27], rabies virus glycoprotein [28] and Japanese encephalitis virus (JEV) envelope protein [29].

In the present study, recombinant baculoviruses expressing WNV prM and E protein were constructed with or without pseudotyping with VSV/G. Protein expression was characterized in transduced mammalian cells and its immunogenicity was investigated in a mouse model. The results showed that direct immunization with the recombinant baculoviruses induced a higher level of WNV-specific antibody, neutralizing antibody and gamma interferon compared with E-DIII protein vaccine, suggesting that the recombinant baculoviruses expressing WNV prM and E protein could be developed as new vaccine candidates for WNV.

## Results

### Construction of recombinant baculoviruses expressing WNV protein

**Figure 1:**
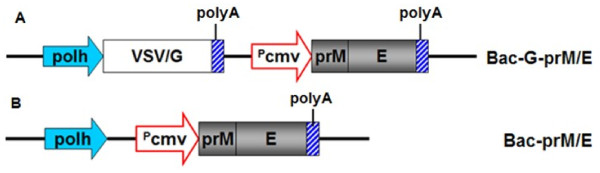
**Schematic presentation for the construction of the recombination baculoviruses used in this study.** Baculovirus transfer plasmids were derived from pFastBac1 and pFastBac-VSV/G as described in Materials and Methods. The expression of prM/E genes of WNV is driven by the CMV immediate-early promoter (P_CMV_ indicated by a red arrow). VSV/G, the glycoprotein of vesicular stomatitis virus, is driven by polyhedrin promoter of baculovirus (polh); polyA, polyadenylation signal.

Recombinant baculovirus has been used as gene-delivery vehicles for transient expression of recombinant proteins in mammalian cells. In the present study, recombinant baculoviruses Bac-prM/E and Bac-G-prM/E expressing WNV prM and E gene under the control of the cytomegalovirus immediate early promoter were constructed (Figure 1).

**Figure 2:**
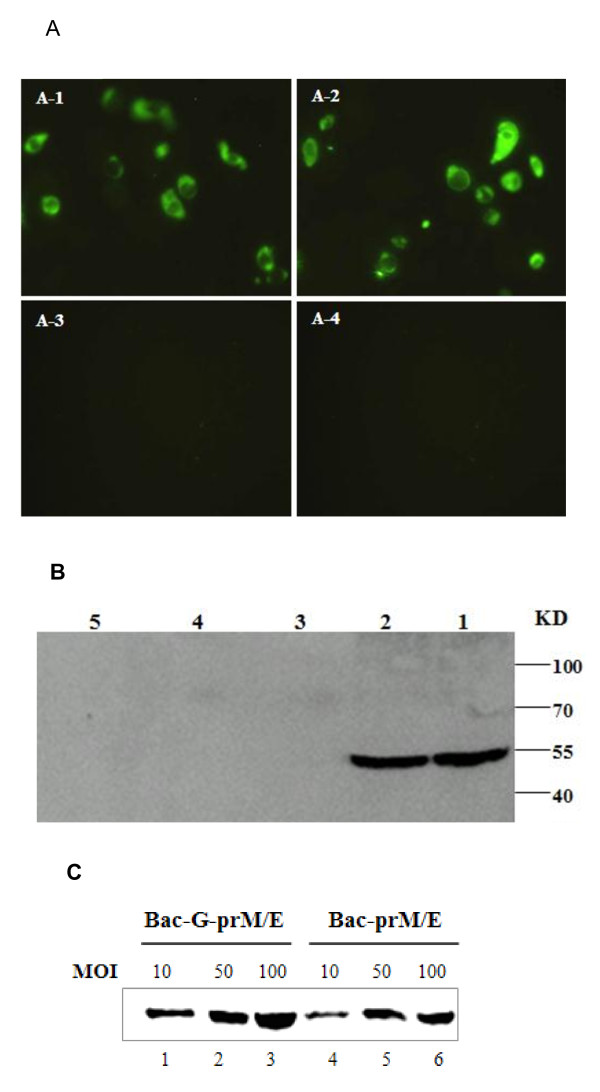
**Expression of WNV protein in baculovirus-transduced cells.(A)** BHK-21 cells were transduced with Bac-G-prM/E (A-1), Bac-prM/E (A-2), Bac-G-EGFP (A-3), or Bac-EGFP (A-4), at a MOI of 100. At 48h post-transduction, the cells were fixed and immunofluorescence assay was conducted using monoclonal antibodies against WNV E-DIII protein, followed by fluorescein isocyanate-conjugated goat anti-mouse IgG. Fluorescent images were taken with a fluorescence microscope (400× original magnification). **(B)** BHK-21 cells were transduced with Bac-G-prM/E (lane 1), Bac-prM/E (lane 2), Bac-G-EGFP (lane 3), or Bac-EGFP (lane 4). Total cell lysates were prepared at 48 h post-transduction and subjected to Western blotting as described in Materials and Methods. Cell lysates from mock-transduced (lane 5) BHK-21 cells were used as a negative control. The positions of molecular size standards (in kilodaltons) were indicated. **(C)** Comparison of the transduction efficiency of Bac-G-prM/E and Bac-prM/E. BHK-21 cells were transcuced with Bac-G-prM/E (lane1-3) or Bac-prM/E (lane 4–6) at MOIs of 10, 50, and 100 and processed for Western blotting analysis.

In order to investigate the transduction efficiency of the constructed recombinant baculoviruses Bac-prM/E and Bac-G-prM/E. BHK-21 cells were transduced with the constructed baculoviruses or control baculovirus (Bac-G-EGFP or Bac-EGFP) at a multiplicity of infection (MOI) of 100. An indirect immunofluorescence assay and Western blotting were performed 48h post-transduction to measure the level of WNV protein expression in mammalian cells. As shown in Figure 2A, significant fluorescence was detected in Bac-prM/E- and Bac-G-prM/E- transducd BHK-21 cells, whereas it was not detected in the Bac-EGFP- or Bac-G-EGFP- transduced cells. A protein band of approximately 55 KDa was consistently observed in recombinant baculovirus-transduced BHK-21 cell lysates as detected by Western blotting (Figure 2B). BHK-21 cells were also transduced with recombinant baculoviruses at different MOIs (10, 50 and 100) and E protein was compared by Western blot analysis. As shown in Figure 2C, the cells transduced with Bac-G-prM/E showed a darker band than cells transduced with Bac-prM/E and the protein expression was increased in a dose-dependent pattern in cells transduced with both viruses.

### Characterization of WNV-specific antibodies elicited by recombinant baculoviruses in mice

**Figure 3:**
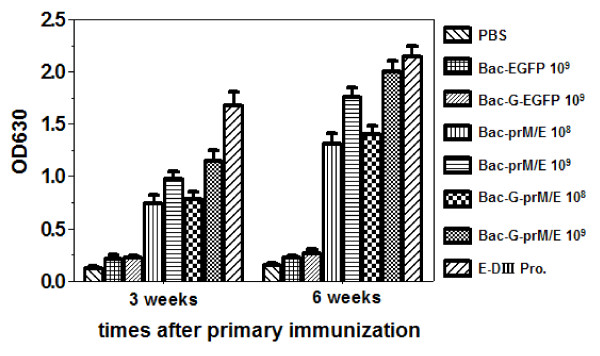
**WNV-specific ELISA antibody titers in mice immunized with recombinant baculoviruses.** Mice were intramuscularly vaccinated with recombinant baculovirus Bac-G-prM/E or Bac-prM/E, or control virus, while E-DIII protein vaccine used as a positive control (for details, see materials and methods, mouse immunization). Serum samples were collected at 3 and 6 weeks after the fist immunization. Presence of antibodies was analyzed by ELISA at a 1:500 serum dilution. The data are expressed as average titers per group (n = 6) ± SD.

Our previous studies have demonstrated the potential of using baculovirus as a vaccination platform [29]. To determine whether the recombinant baculoviruses expressing prM/E proteins could induce WNV-specific humoral immune responses *in vivo*, BALB/c mice were immunized intramuscularly with 10^9^ or 10^8^ PFU/mouse of recombinant baculovirus. Serum samples were collected at 3 and 6 weeks after the primary immunization. Then the samples were diluted 1: 500 and specific IgG responses were determined by an indirect ELISA using E-DIII protein as coating antigen. At 3 weeks, the antibody titers reached a detectable level in all the vaccinated groups except the group immunized with the Bac-G-EGFP, Bac-EGFP (control virus) and PBS, and a further increase in antibody titers were observed at 6 weeks after primary immunization (*p*<0.001) (Figure 3). Mice immunized with 10^9^ PFU of Bac-G-prM/E produced significantly higher antibody titer than mice received 10^8^ PFU of Bac-G-prM/E (*p*<0.01). Similar result was also observed in Bac-prM/E-vaccinated group. Interestingly, mice immunized with Bac-G-prM/E developed higher antibody titer than mice immunized with Bac-prM/E at the same dosage, but there was no statistical significance (*p*>0.05). Immunization with 50μg/mouse of E-DIII proteins induced highest antigen-specific antibody titer. As expected, all vaccinated groups produced significantly higher WNV-specific antibody titers than mice vaccinated with control viruses (*p*<0.001).

In order to further study whether recombinant baculovirus-immunized mice induce WNV-specific neutralizing antibodies, serum samples were tested by PRNT. As shown in Table 1, mice immunized with 10^8^ or 10^9^ PFU of Bac-G-prM/E or Bac-prM/E developed mean neutralizing antibody titers of 1:16 and 1:8 or 1:11 and 1:5 at 3 weeks after primary immunization, which increased further to 1:44 and 1: 22 or 1:30 and 1:12 at 6 weeks, respectively. Although WNV-specific neutralizing antibodies were detectable in mice immunized with 50μg of E-DIII protein, the mean neutralizing antibody titer was 1:8 at 6 weeks, which was significantly lower than the groups inoculated with 10^9^ PFU of Bac-G-prM/E and Bac-prM/E (*p*<0.05). As expected, no neutralizing antibodies were detected in sera from mice immunized with control virus or PBS. In addition, we also determined whether the antibodies can efficiently neutralize heterologous Japanese encephalitis virus (JEV)-p3 strain. Little or none anti-JEV cross-neutralizing antibodies were detected in sera collected at 3 weeks after the first immunization. After booster immunization, anti-JEV cross-neutralizing antibody titers increased to 1:10, which were lower than those against WNV. These studies indicate that recombinant baculoviruses immunized mice developed humoral immune responses evidenced by WNV-specific neutralizing antibodies and partial anti-JEV cross-neutralizing antibodies.

**Table 1:** Neutralizing antibody titers in mice immunized with recombinant baculoviruses (mean values of groups of 4 mice)

**Vaccine**	**Anti-WNV**^**b**^		**Anti-JEV**^**c**^	
	**3 weeks**	**6 weeks**	**3 weeks**	**6 weeks**
Bac-G-prM/E 10^9^	16	44	4	10
Bac-G-prM/E 10^8^	8	22	< 2	6
Bac-prM/E 10^9^	11	30	< 2	7
Bac-prM/E 10^8^	5	12	< 2	4
E-DIII pro.	4	8	< 2	< 2
Bac-G-EGFP 10^9^	< 2	< 2	< 2	< 2

Samples were taken three weeks and six weeks after the primary immunization.^a^

^a^ plaque-reduction neutralization tests (PRNTs);

^b^ WNV (NY99 strain)-specific neutralizing antibodies; ^c^ JEV(P3 strain)-specific neutralizing antibodies; < 2, not detected.

### WNV-specific cellular immune responses elicited by recombinant baculoviruses in mice

**Figure 4:**
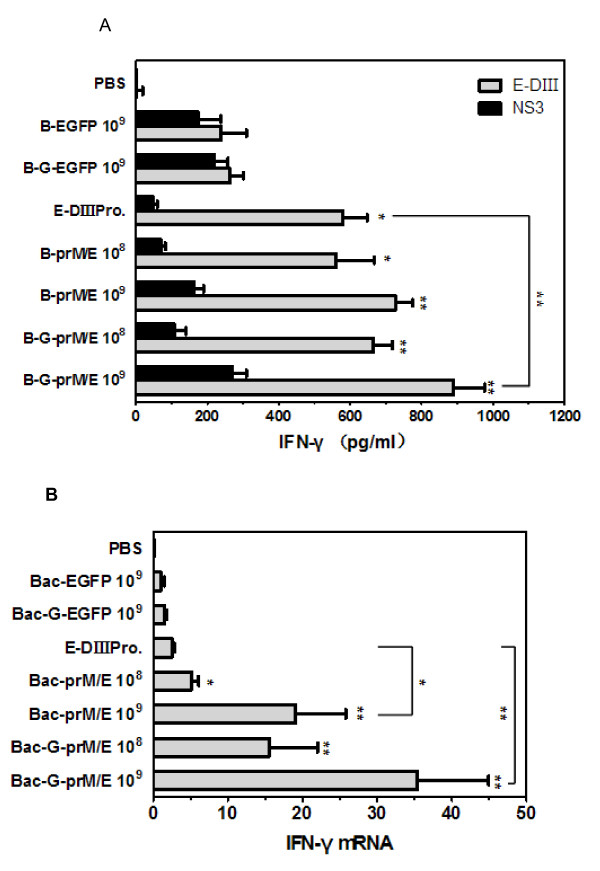
**IFN-γ responses induced by immunization with recombinant baculoviruses.(A)** Production of IFN-γ in the supernatant of splenocytes harvested from immunized mice after *in vitro* restimulation was determined by ELISA. Splenocytes of immunized mice were isolated 6 weeks after the primary immunization and incubated *in vitro* with E-DIII protein or NS3 protein for 72h. Data represent the mean concentrations of IFN-γ in the supernatant ± SD of the three independent experiments. **(B)** IFN-γ relative gene expression in splenocytes harvested from immunized mice after *in vitro* restimulation with or without E-DIII protein for 24h. RNA was extracted and subjected to RT-PCR. Relative quantity of IFN-γ mRNA expression was determined by relative quantitative real-time PCR using β-actin gene as housekeeping gene. The bars in graph denote the mean relative quantity of IFN-γ mRNA ± SD observed from three mice per group, each performed in triplicate. *, *p*<0.05; **, *p*<0.01 compared to the value for 10^9^ PFU of Bac-G-EGFP (control virus)-immunized group.

To further address whether the recombinant baculoviruses can elicit cell-mediate immune responses in mice, mice were immunized with the recombinant baculoviruses and sacrificed at 3 weeks after the booster immunization. Splenocytes were prepared from the immunized mice and restimulated *in vitro* with E-DIII protein. IFN-γ production was measured by ELISA. As a negative control, IFN-γ production was also measured upon stimulation of splenocytes with NS3 protein of JEV. Mice immunized with 10^9^ PFU, 10^8^ PFU of Bac-G-prM/E and Bac-prM/E or 50μg of E-DIII protein showed significantly higher levels of IFN-γ than mice given the control virus (10^9^ PFU of Bac-G-EGFP) (Figure 4A). In particular, IFN-γ level detected in splenocytes of mice inoculated with 10^9^ PFU of Bac-G-prM/E was significantly higher than that in splenocytes of mice immunized with E-DIII protein (*p*<0.01). It was also found that mice immunized with Bac-G-prM/E developed higher amount IFN-γ than mice receiving Bac-prM/E, but it was not statistically significant. Moreover, IFN-γ production in Bac-G-prM/E- or Bac-prM/E-inoculated group was significant lower when NS3 protein was used as stimulation antigen and there was no significant difference among Bac-G-prM/E, Bac-prM/E, and control virus immunized group, suggesting WNV specific IFN-γ production (Figure 4A).

To validate this observation, mRNA level of IFN-γ produced in splenocytes was further analyzed by real-time PCR in parallel with the housekeeping gene. Similar to the results of IFN-γ determined by ELISA, the mean relative level of IFN-γ mRNA expression showed significant difference between Bac-G-prM/E- or Bac-prM/E-immunized mice and Bac-G-EGFP- or E-DIII protein-inoculated mice (Figure 4B). All these results demonstrate that immunization with recombinant baculoviruses can stimulate cellular immune responses.

### Recombinant baculoviruses induce inflammatory cytokines in mice

**Figure 5:**
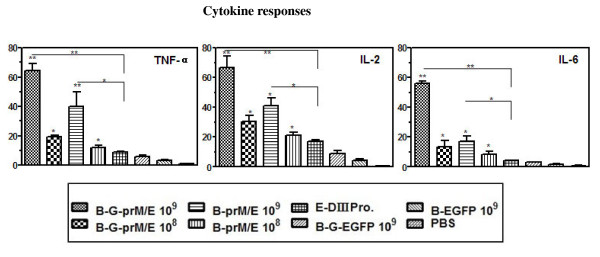
**Analysis of inflammatory cytokine production following immunization with baculoviruses.** Real-time PCR was conducted for three cytokines, TNF-α, IL-2 and IL-6. Total RNA was obtained as described in Figure 4. The graphs obtained for the expression of TNF-α gene, IL-2 gene and IL-6 gene, respectively. Data are presented as means ± SD (n = 3). *, *p*<0.05; **, *p*<0.01 compared to the value for 10^9^ PFU of Bac-G-EGFP (control virus)-immunized group.

To further understand the mechanism associated with induction of host immune response upon baculovirus inoculation, the expression profile of inflammatory cytokines was monitored. Splenocytes from immunized mice were restimulated *in vitro* and then mRNA levels of three inflammatory cytokines, including tumor necrosis factor alpha (TNF-α), interleukin-6 (IL-6), and interleukin-2 (IL-2), were detected by real-time PCR. As shown in Figure 5, the mean relative mRNA levels of these inflammatory cytokines in the group immunized with Bac-G-prM/E or Bac-prM/E were significantly higher than in the control group. Moreover, mice immunized with 10^9^ PFU of Bac-G-prM/E or Bac-prM/E produced significantly higher amount of these inflammatory cytokines than mice immunized with E-DIII protein (*p*<0.05). These suggest that a strong inflammatory response with a complex pattern of inflammatory cytokines may contribute to the intrinsic immunogenic properties of the inoculated baculoviruses.

## Discussion

WNV continues to present public health threat to the western hemisphere, yet, there is neither effective therapy nor vaccines available for humans. Thus developing a safe and effective vaccine is a priority. Several immunization strategies have been described, some of which utilize vectored vaccines expressing WNV glycoprotein E resulted in 80%-100% protective immunity in animal model [30-34]. In this present study, attempts were made by using recombinant baculovirus approach for WNV vaccine development since baculovirus exhibit low toxoxicity in mammalian cells and can induce interferon production [24]. Two recombinant baculoviruses, Bac-G-prM/E and Bac-prM/E, expressing the major immunogenic protein, glycoprotein E, were constructed,and the humoral and cellular immune responses were determined in mice. WNV-specific humoral (WNV-specific antibodies and neutralizing antibodies) and cellular immunity (WNV-specific IFN-γ and inflammatory cytokines) were induced in mice inoculated with recombinant baculoviruses. These data are in concordance with previous studies that showed robust humoral and cellular immune responses against PRRSV, PCV, or JEV by recombinant baculoviruses [29].

It has been controversial as to the relative role of the humoral versus cellular immune responses in the WNV-protective immunity. High antibody titers are believed to protect animals against flavivirus infections through direct neutralization of receptor binding, inhibition of viral fusion, and/or complement-mediated viral clearance [35,36]. Neutralizing antibody titers of >1:10 were reported to be sufficient to afford protection in humans against JE [37]. Schneeweiss et al. also reported that mice immunized once with pT-WNV-E produced neutralizing antibodies titer of 1:16 which can protect 100% of mice from WNV challenge [38]. However, others have argued that cellular immune responses such as robust anti-CD8^+^ T cell responses are essential for clearing WNV [39,40]. In our study, the antibody responses were evaluated in mice immunized with recombinant baculoviruses. Majority of mice immunized with two dosages of Bac-G-prM/E or Bac-prM/E developed neutralizing antibodies titers of >1:16 (Table 1). Furthermore, these baculovirus-derived vaccines can also induce anti-JEV cross-neutralizing antibodies although the titers of the antibodies were lower than antibodies against WNV. These results strongly demonstrate that the antibody response induced by WNV protein could partially cross-neutralize JEV, as has been reported in previous studies [41]. It is reasonable to anticipate that immunization with these recombinant baculoviruses expressing WNV antigens will not only provide protection against WNV infection, but also provide partial protection against JEV infection. Cellular immunity such as IFN-γ production and inflammatory cytokines responses were also determined in mice after immunization with these baculoviruses since these cytokines play a critical role in directing the cell-mediated immune responses [42]. Bac-G-prM/E- or Bac-prM/E-immunized mice produced higher levels of IFN-γ than any other group (Figure 4) and promoted the release of inflammatory cytokines, including tumor necrosis factor alpha (TNF-α), interleukin-6 (IL-6), and interleukin-2 (IL-2) (Figure 5). In this case, secretion of IFN-γ, TNF-α, or IL-2 are indicative of a Th1 response, whereas a Th2 response is characterized by induction of IL-6 [43]. These indicate that the recombinant baculoviruses can stimulate both humoral and cellular immune responses which are essential to control dissemination of infection and to inhibit the entry of virus into brain.

Our results also demonstrate that Bac-G-prM/E exhibited better immunogenicity than Bac-prM/E, as indicated by the higher levels of antibody titers, IFN-γ and inflammatory cytokines production. It is highly probable that the expression of VSV/G was associated with enhancement of the immune responses by augmenting transduction efficiency of baculovirus into mammalian cells [20]. In addition, the VSV/G-modified baculovirus exhibited greater resistance to animal serum inactivation than the unmodified baculovirus [20], which may contribute to the better immunogenicity of Bac-G-prM/E.

It has been reported that recombinant E-DIII protein induces high neutralizing antibody titers, as well as protection against lethal WNV and partial protection against lethal JEV [41]. Therefore, the recombinant E-DIII protein of WNV expressed in *E.coli* was used as a positive control in the present study. However, intramuscular injection of mice with E-DIII protein elicited lower levels of neutralization antibody titers than mice immunized with the recombinant baculoviruses even at a low dosage (10^8^ PFU/mouse) (Table 1), although the total IgG level was high (Figure 3). This could be due to differences in experimental designs such as the route of immunization, with or without adjuvant, and mouse strain. In addition, it has been shown that E-DIII protein immunization elicits low level of neutralizing antibodies with relatively high IgG responses [38], which is consistent with our results. It is noticed that E-DIII protein also induced lower levels of cellular immune response than recombinant baculoviruses, since E protein expressed by recombinant baculovirus contains more T cell epitopes than its domain III [40], and the baculovirus augment cellular immunity.

Baculovirus has been shown to possess a strong adjuvant activity and to promote humoral and cellular immune responses for foreign antigens, maturation of dendritic cells, and production of inflammatory cytokines and IFN [24]. The transduction of macrophages *in vitro* by baculovirus led to the induction of significant levels of IL-6 and TNF-α [44]. It has been proposed that baculovirus genome, especially its CpG motifs, could be recognized by macrophages and DCs. Furthermore, baculoviruses enter into the cells through mannose receptor (MR)-mediated endocytosis or phagocytosis, leading to the secretion of inflammatory cytokines through a MyD88/TLR9-dependent signaling pathway [23,24]. However, Chen et al. reported that recombinant baculoviruses are able to induce a robust secretion of inflammatory cytokines through a TLR3-dependent pathway [45]. In our study, induction of a measurable IFN-γ and inflammatory cytokines, including TNF-α, IL-6, and IL-2 by control baculovirus (Bac-G-EGFP and Bac-EGFP) was observed, demonstrating that this virus can function as a stimulator for production of cell-mediate immunity in an antigen- independent manner (Figure 4). Similar results have been reported previously that injection with control baculovirus is capable of inducing IFN-γ responses [26,27,29]. This is probably due to direct interaction of baculovirus with dendritic cells and macrophages in the spleen. Since it has been reported that splenic dendritic cells and macrophages play a central role in baculovirus-induced inflammatory responses in mice [44]. These baculovirus- induced non-specific inflammatory responses raise concerns that whether they will compromise the use of baculovirus vectors for *in vivo* gene therapy in humans and animals. However, Bac-G-E2 inoculated mice released inflammatory cytokines as early as 6h post-injection, and returned to background levels by 48h post-injection [21]. Furthermore, there was no cytokine-induced acute toxicity or mortality in baculovirus-immunized mice, which suggests that non-specific inflammatory responses induced by baculovirus vectors may be minimized by host. Nevertheless, more investigations are needed to ensure the safety of baculovirus vectors.

The protection is an important index to evaluate the efficiency of vaccine. However, the ability of our recombinant baculoviruses to protect against lethal challenge of WNV was not determined in consideration of the safety problem since WNV has not been found in China. Nevertheless, the neutralizing antibodies and the cellular immune responses induced by recombinant baculoviruses may provide the basis for protection. Immunization with baculovirus expressing JEV antigens have been shown to confer protection against JEV challenge, demonstrating that cellular immunity plays a crucial role against JEV infection [29]. Thus it is well supported that recombinant baculoviruses expressing WNV prM/E proteins could provide protective immunity against WNV challenge.

## Methods

### Cells, plasmids and virus

Spodoptera frugiperda 9 (Sf9) cells were propagated at 28°C in Grace’s media (Invitrogen, Carlsbad, CA, USA) supplemented with 10% FBS, 100 μg/ml streptomycin and 100 IU/ml penicillin. BHK- 21 cells, used for transduction and neutralization tests, were grown and maintained in Dulbecco’s modified Eagle’s medium (DMEM; Invitrogen, Carlsbad, CA, USA) supplemented with 10% heated-inactivated fetal bovine serum (FBS), 100 μg/ml streptomycin and 100 IU/ml penicillin, at 37°C with 5% CO_2_.

The prM and E genes region (GenBank accession number DQ211652) corresponding to nt 466–2469, derived from a North American NY99 strain of WNV, were synthesized and cloned into pcDNA3.1 vector backbone, to generate plasmid pcDNA3.1-prM/E. The construction of baculovirus transfer vector, pFastBac-VSV/G, in which the VSV/G gene is under the control of the polyhedrin promoter of pFastBac^TM^1 (Invitrogen), was described previously [29]. Bac-G-EGFP is a pseudotype baculovirus containing a CMV promoter that controls the expression of EGFP [29] and was used as a control strain of the baculovirus.

### Protein and monoclonal antibodies

WNV envelope protein (E protein) domain III (the sequence corresponding to nt 1858–2211) was expressed in *E.coli* as an inclusion body, and then purified and refolded in appropriate buffer [46]. Protein concentrations were determined at an absorbance of 280 nm by spectrophotometry. Anti-E-DIII monoclonal antibody (MAb) was produced from the mice immunized with E-DIII protein.

### Construction of recombinant baculoviruses

To generate the recombinant transfer plasmid pFastBac-G-prM/E, the DNA fragment containing the prM/E expression cassette, which was controlled under the CMV promoter, was released from pcDNA3.1-prM/E and inserted into pFastBac-VSV/G. To generate the recombinant transfer plasmid pFastBac-prM/E the same prM/E expression cassette was digested and clone into pFastBac1 vector. The recombinant baculoviruses Bac-G-prM/E and Bac-prM/E were subsequently generated by using Bac-to-Bac system (Invitrogen) following the manufacturer’s instructions. The resultant viruses were further amplified by propagation in Sf9 cells and purified as described [25]. The virus pellet was resuspended in phosphate-buffer saline (PBS, PH7.4) and infectious titers were determined by a plaque assay as described in the Bac-to-Bac system, and then stored in aliquots at −80°C until needed.

### Detection of E protein in Baculovirus-transduced cells

The transduction procedure was performed as described previously with minor modifications [27,29]. BHK-21 cells were seeded into six-well plates at concentration of 1.5 × 10^5^ cells/well. At approximately 70-80% confluence, culture medium was removed and cells were washed three times with PBS and incubated with baculoviruses for 4h at 28°C. After removal of the inoculum, fresh medium was added and cultures incubated at 37°C for 48h. Duplicate wells were processed in parallel for indirect immunofluorescence assay and Western blotting. At 48h after transduction, the cells were fixed with absolute methanol and processed for the indirect immunofluorescence assay using monoclonal antibodies (MAb) against WNV E-DIII protein, followed by fluorescein isocyanate-conjugated goat anti-mouse immunoglobulin (Ig) G. For Western blot analysis, transduced cells were collected at 48h post-transduction and lysed in SDS sample buffer. Cell extracts were separated by sodium dodecyl sulfate −10% polyacrylamide gel electrophoresis (SDS-PAGE) and electroblotted onto a nitrocellulose membrane. The nonspecific antibody binding sites were blocked with 1% bovine serum albumin (BSA) in TBST buffer (10mM Tris–HCl PH 8.0, 150mM Nacl, and 0.05% Tween-20), and finally the membrane were reacted with anti-E-DIII Mab to detect the expressed E protein.

### Mouse immunization

Female BALB/c mice (6–8 week old) were purchased from the Animal Centre, Institute of Medicine, Hubei province, China. Mouse studies were performed according to the guidelines of this institution (No. 00020502) and all experimental protocols were approved by the Research Ethics Committee of College of Veterinary Medicine, Huazhong Agricultural University, Hubei, China. Then the mice were randomly divided into eight groups (6 mice per group). Two groups were injected intramuscularly (i.m.) with 100μl of PBS containing 1 × 10^8^ or 1 × 10^9^ PFU of Bac-G-prM/E and another two groups were immunized with Bac-prM/E. The other three groups were injected intramuscularly with 100μl of PBS containing 1 × 10^9^ PFU of Bac-G-EGFP, Bac-EGFP, and 100μl of PBS containing 50μg of E-DIII protein, respectively. The last group was used as a negative control by intramuscularly injecting 100μl of PBS. Booster immunizations were identically performed 3 weeks later. Serum samples were collected from the tail vein of 6 individual mice before the second injection and from the retro-orbital plexus at 6 weeks after primary immunization and then store at −20°C for serological tests. At 6 weeks after the primary immunization, three mice of each group were euthanized, and splenocytes were harvested for immunological assay.

### Antibody assay

Induction of E protein-specific antibody was determined by using indirect enzyme linked immunosorbent assay (ELISA). Microplates were coated with E-DIII protein (20ng/well) overnight at 4°C [38]. After blocking for 1h at 37°C with PBST (2% bovine serum albumin, PBS, 0.05% Tween 20) to avoid nonspecific binding, serum diluted in PBST (1:500) were added to each well and incubated at 37°C for 1h. After washing with PBST, bounded proteins were detected with HRP conjugated with goat anti-mouse IgG (Boster, China). Serum antibody titer was monitored versus optical density values. The endpoint titers of the neutralizing antibodies against WNV and JEV were determined by plaque-reduction neutralization tests (PRNTs) using WNV strain NY99 and JEV strain P3, in biosafety level–3 conditions,as described earlier [47]. Briefly, serum samples were heat-inactivated for 45min at 56°C and then serially diluted and mixed with an equal volume containing 100 PFU of virus. The mixtures were incubated for 1h at 37°C and then inoculated onto BHK-21 cell monolayers in 12-well plates. After adsorption for 1h at 37°C, the wells were overlaid with medium containing 1.5% carboxymethyl cellulose, and the plates were incubated in a 5% CO_2_ incubator at 37°C for 3–4 days for plaque formation, then fixed with 10% formalin in PBS. Plates were washed and stained with 0.01% crystal violet. The percent plaque reduction was calculated relative to virus controls without serum. The PRNT titers were given as the reciprocal of the highest serum dilutions which resulted in > 50% reduction of the plaques.

### IFN-γ detection

Mouse splenocytes were prepared from immunized mice and resuspended in RPMI 1640 (Invitrogen, Carlsbad, CA, USA) supplemented with 10% FBS, 2mM _L_-glutamine, 100μg/ml streptomycin and 100 IU/ml penicillin. Splenocytes (1 × 10^6^/ml) were cultured in 24-well plates at 37°C in the presence of 20μg/ml WNV E-DIII or JEV NS3 protein. After 72h incubation, culture supernatant was harvested and the presence of IFN-γ was measured with commercial mouse IFN-γ immunoassay ELISA kits (eBioscience Inc., San Diego, CA) according to the manufacture’s guidelines. The concentrations of IFN-γ in the samples were calculated from the standard curves.

### Analysis of cytokine mRNA expression by Real-time PCR

Mouse splenocytes (1 × 10^6^/ml) were maintained in 24-well plates for 20h at 37°C in the presence of 5% CO2, with or without 20μg/ml E-DIII protein. Total RNA from each immunized group was extracted using Trizol (Invitrogen) in accordance with the manufacture’s guidelines. RNA concentrations were determined by OD260 measurements and 1μg of RNA was reverse transcribed using the Rever Tra Ace^TM^ kit (ToYoBo, China) in a 20μl reaction mixture. Afterward, the cDNA pool (0.5μl) was amplified in a 25μl reaction mixture containing SYBR^TM^ Green real-time Master Mix (ToYoBo, China) and 0.2μM each of the forward and reverse gene-specific primers (listed in Table 2), aliquoted into 96-well plates (Axygen, CA, USA), and then sealed with optical sealing tape (Axygen, CA, USA). Real-time PCR was performed in triplicate by using an Applied biosystems 7500 Real-time PCR System and universal cycle conditions (2min at 50°C, 10min at 94°C, 40 cycles of 15s at 94°C and then 1min at 60°C). House keeping gene β–actin was measured in parallel, which was used as the internal control. In order to evaluate the expression of the target gene by relative quantity, the threshold cycle (Ct) of the sample of interest was compared to the Ct generated by a reference sample referred to as the calibrator (a sample from nonstimulated splenocytes). Cytokine gene expression was normalized to β–actin expression by the subtraction of Ct to provide ΔCt values. The ΔΔCt was calculated as the difference between ΔCt values for stimulated and nonstimulated splenocytes (the calibrator). Thus, the relative difference in cytokine expression between stimulated and nonstimulated cells was calculated as fold change by using equation 2^-ΔΔCt^.

**Table 2:** Relative quantitative real-time PCR primers for targeted transcripts mRNAs

**Targeted transcripts**^**a**^	**Primer sequence (5’to 3’)**		**Conditions**
	**Forward**	**Reverse**	
β-actin^b^	CACTGCCGCATCCTCTTCCTCCC	CAATAGTGATGACCTGGCCGT	50°C 2min; 94°C 10min 40 cycles (94°C 15s then 60°C 1min)
IFN-γ	TCAAGTGGCATAGATGTGGAAGAA	TGGCTCTGCAGGATTTTCATG	
TNF-α	TGTCTCAGCCTCTTCTCATTCC	TTAGCCCACTTCTTTCCCTCAC	
IL-2	CATTGACACTTGTGCTCCTTGT	TCCTGTAATTCTCCATCCTGCT	
IL-6	CATGTTCTCTGGGAAATCGTG	TCCAGTTTGGTAGCATCCATC	

^a^ Mouse derived targeted transcripts. ^b^House keeping gene for internal control.

### Statistical analysis

Where specified, an unpaired *t*-test was used to compare the humoral and cellular immune responses between the different groups. All comparisons were made using two-tailed and *P*-values of 0.05 or less were considered statistically significant.

## Competing interests

The authors declare that they have no competing interests.

## Authors’ contributions

BZ performed the majority of experiments and involved in manuscript preparation. JY participated in editing of the manuscript. JY, PL, RJ, and XY participated part of the experiments. FZ, HC and SC conceived of the study, participated in its design and coordination, and revised the manuscript. All authors read and approved the final manuscript.
